# Rejection sensitivity mediates the interparental conflict and adolescent Internet addiction: School connectedness as a moderator

**DOI:** 10.3389/fpsyg.2022.1038470

**Published:** 2022-10-28

**Authors:** Zhiyuan Tao, Xiuli Zhao, Zhenhai Wang, Chengfu Yu, Wei Zhang

**Affiliations:** ^1^Center for Studies of Psychological Application, School of Psychology, South China Normal University, Guangzhou, China; ^2^School of Education, South China Normal University, Guangzhou, China; ^3^Department of Psychology and Research Center of Adolescent Psychology and Behavior, School of Education, Guangzhou University, Guangzhou, China; ^4^Academy of Art and Design, Guangdong AIB Polytechnic, Guangzhou, Guangdong, China

**Keywords:** Interparental conflict, rejection sensitivity, school connectedness, early adolescents, Internet addiction

## Abstract

Internet addiction (IA) is a growing social problem with negative mental and social outcomes; the present study examined whether rejection sensitivity mediates the relationship between interparental conflict and adolescent IA and the moderating role of school connectedness. One thousand and seven adolescents (51.84% females; *Mean_age_* = 13.17; *SD* = 0.69) anonymously completed questionnaires to assess interparental conflict, school connectedness, rejection sensitivity, IA, and demographic information. The model results showed that: (1) the positive association between interparental conflict and adolescent IA was partially mediated by rejection sensitivity; (2) this indirect link was moderated by the school connectedness and was stronger for adolescents with high school connectedness. The results provide support for the attachment theory that high interparental conflict could increase adolescents’ rejection sensitivity, and high school connectedness plays a double-edged role that adolescents show more rejection sensitivity while reporting high interparental conflict and high school connectedness.

## Introduction

Adolescents are active Internet users in China. According to the China Internet Network Information Center, as of November 2020, the number of juvenile Internet users increased to 183 million, and the Internet penetration rate was 94.9%, up 1.8% points from 2019 (CNNIC, 2021).

Although the Internet can be helpful in many aspects of adolescents’ lives, there is also concern that youth spend too much time online. Internet addiction (IA) is defined as an impulse-control disorder that results in an inability to control Internet use and results in problems in school, work, and social relationships (Spada, 2014).

Multiple studies have found the negative consequences of IA, IA was significantly associated with individual fatigue, depression, anxiety, headache, and other mental and physical health problems (Bener, 2017; Upadhayay and Guragain, 2017; Chen et al., 2020). Serious IA will seriously affect daily life for both teenagers and adults, such as low tolerance of stress, and scholastic or occupational impairment (Young and De Abreu, 2011; Jun and Choi, 2015). Compared with adults, the existing research supports that adolescents are more susceptible to IA because of their poor self-control (Spada, 2014). Adolescents who show characteristics of IA will report less novelty seeking in reality that they may generate more cyber fantasies and replace them with reality (Mittal et al., 2013); besides, IA brings more reward dependence and impulsivity to adolescents which makes them more vulnerable to other addictive behaviors (Leeman et al., 2019). In addition to causing behavioral problems, IA is also a nonadaptive way for adolescents to deal with negative emotions, and through such negative reinforcement, the degree of IA in adolescents may be further deepened (Pettorruso et al., 2020).

In recent years, researchers have begun to explore the risk factors of IA to alleviate this social problem (Wang et al., 2019; He et al., 2021; Wang M. et al., 2021). A meta-analysis demonstrated that, as a family risk factor, interparental conflict is deeply connected to IA (Van Eldik et al., 2020). Because that adolescents living in a conflict family environment are at high risk for serious mental problems, the condition “child affected by parental relationship distress” was introduced into DSM-5 (Harold and Sellers, 2018). Attachment theory (Bowlby, 1982) points out that while children perceive interparental conflict, their sense of security may be threatened and may shape an insecure attachment style (Liu et al., 2020; Wang M. et al., 2021). In this context, adolescents are more likely to suffer from depressive symptoms, and other internal adverse outcomes (Harold and Sellers, 2018; Ekas and Kouros, 2020). Except for these mental adverse outcomes, it has been proved that there is a strong association between interparental conflict and adolescent IA (Yang et al., 2016; Zhou et al., 2017; Smith et al., 2019; Li et al., 2021).

Interparental conflict may damage adolescents’ self-control, which will likely carry over to IA (Willems et al., 2018). Besides, the interparental conflict will create a risky environment in which youth are distracted from positive activities and consume their energy on Internet, drinking, and delinquency (Lu et al., 2020). The perception of strong conflicts between the parents of adolescents may threaten their inner security and reduce supportive resources Huang et al., 2021; consequently, adolescents feel helpless and try to turn to the Internet as the most favorable place to relieve stress and provide emotional support (Wang L. X. et al., 2021). In addition, IA may be an adaptation and an adverse coping strategy for depression and anxiety caused by interparental conflict (Rosser-Liminana et al., 2020). Then, Internet use may provide a chance to escape from these psychological and emotional burdens (Ko et al., 2015).

In general, the influences of interparental conflict on adolescent IA are complicated, and high interparental conflict may positively relate to various types of IA (e.g., gaming and social media; Wang, 2021) through internal emotional and cognitive mechanisms. Studies have also emphasized the importance of exploring the subtypes of IA because the Internet is just a carrier, and the content of Internet addiction may be more important. However, under the premise that parental conflict may lead to multiple IA behaviors, we think it is necessary to directly study IA (Baggio et al., 2022). It can be seen that all the related studies above demonstrate the positive association between interparental conflict and IA, and this relationship appears to be strong. Thus, the underlying mechanisms that could account for this link (i.e., mediating mechanisms) and buffer it (i.e., moderating mechanism) become quite important.

### Rejection sensitivity as a mediator

Rejection sensitivity is characterized by a fear of, and emotional and behavioral overreaction to minimal or ambiguous rejection clues cause by their anticipatory rejection anxiety, people who show high rejection sensitivity may easily feel humiliation and betrayal from others (Downey and Feldman, 1996). They have the disposition to misinterpret and show hypervigilance toward ambiguous social clues (Ehrlich et al., 2015). This behavior pattern can cause people to feel separated from others and can prevent them from having their psychological needs met, leaving them at increased risk of IA (Chang et al., 2014). According to the attachment theory (Bowlby, 1977), all individuals build their internal working models, comprising of core schema, goals, motives, autobiographical memories, and behavioral strategies with the experience that how their caregivers interact with them (Main et al., 1985). Rejection sensitivity was thought to develop from an adverse family environment (Downey et al., 2004), and empirical studies support this opinion that interparental conflict is strongly positively related to adolescents’ rejection sensitivity, it may bring anxiety and anger expectations to adolescents (Rudolph and Zimmer-Gembeck, 2013). And high rejection sensitivity individuals will further form maladaptive schema, emotion dysregulation, low self-esteem, and finally lead to behavioral disorder (Selby et al., 2009; Cardi et al., 2012; Nepon et al., 2020).

At the same time, emotional security theory (Davies and Cummings, 1994) emphasized the importance of the mediating mechanism of emotional insecurity-related problems (e.g., rejection sensitivity) between interparental conflicts and behavioral problems (Lux and Walper, 2019). Cummings et al. (2015) believe that conflicts in the family system may increase individuals’ sensitivity to interpersonal insecurity information; therefore, the development of internal resources for adolescents who have experienced parental conflict is restrained. High rejection sensitivity makes them afraid of rejection and they may spend a lot of psychological resources to pursuit of support and security for a long time, and they may not have sufficient resources to control their behaviors (Mikulincer and Shaver, 2019). Meanwhile, the Internet may be an more advantage zone than reality to compensate for difficulties in their social relationships, relieve anxiety, and meet new partners (McKenna and Bargh, 2000). Research of Farahani et al. (2011) also suggest that people with higher rejection sensitivity had a harder time making friends and were more likely to turn to social networking than the normal one, increasing the risk of social media addiction. In addition, high rejection sensitivity will damage people’s ability for self-regulation, making it difficult to resist Internet use (Molavi et al., 2018). And adolescents with high rejection sensitivity may engage with others on the Internet, including through gaming and social media applications, to avoid the outside world (Weinstein et al., 2016). Therefore, from both theoretical and empirical perspectives, it can be seen that the interparental conflict may destroy the individual’s emotional security and forms an insecure attachment to increase their rejection sensitivity; altogether, rejection sensitivity may be an important characteristic that could mediate interparental conflict and IA.

*Hypothesis 1*: Rejection sensitivity could mediate the association between interparental conflict and adolescent IA.

### School connectedness as a moderator

Although interparental conflict may be a risk factor for children’s problems, there is individual variability in how, and how much, children are affected by it. According to the Ecological Theory, the interaction between school and family system could influence the individual’s developmental trajectory (Bronfenbrenner and Morris, 2006), and the school environment is a vital microsystem for adolescents to develop a secure attachment which could decrease rejection sensitivity (Bergin and Bergin, 2009). Meanwhile, adolescents would begin to rely less on the family but more on school or another context to move from childhood to adolescence and to become independent (Daley, 2019), which enhances the importance of the school environment.

School connectedness is a student’s subjective sense of belonging in a school and having meaningful connections with teachers and other students (Fredricks et al., 2004), and school connectedness has been verified that it is negatively associated with rejection sensitivity (Li et al., 2021). High school connectedness adolescents will report more mental health than others (van Dijk et al., 2020; Zeinalipour, 2021); at the same time, making friends in school will also increase adolescents’ psychological security which could alleviate the uneasiness caused by conflicts in the family and further reduce Internet use (Pontes and Griffiths, 2015; Tian et al., 2019). Besides, school connectedness could reduce depression and anxiety to relieve the individual’s emotional pressure and prevent the individual from distorting the neutral views of others into rejection (Klinck et al., 2019; Lux and Walper, 2019). This evidence shows that school connectedness could be a protective factor to reduce adolescents’ rejection sensitivity. According to the protective-stabilizing model (i.e., protective asset could weaken the impact of risk factors; Fergus and Zimmerman, 2005), we could make a more specific assumption that high school connectedness adolescents may stay in a safety level of rejection sensitivity while low school connectedness adolescents may fall in risk. This is an important research question because when the buffering mechanism of school connectedness is established, the intervention of constructing an adolescent-school connection will be very beneficial for high interparental conflict students. Combined with the Ecological Theory (Bronfenbrenner and Morris, 2006) and the protective-stabilizing model and previous empirical evidence, we believe that school connection, as a more proximal environmental factor, should be able to mitigate the adverse impact of the family system, thereby reducing the individual’s rejection sensitivity, so we give the hypothesis 2.

*Hypothesis 2*: School connectedness could moderate the relationship between interparental conflict and rejection sensitivity.

### The present study

**Figure 1:**
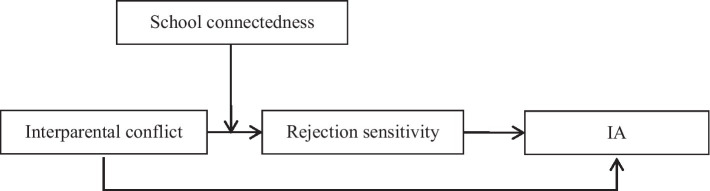
The proposed moderated mediation model.

The present study was guided by the preceding ecological systems theory, attachment theory, and emotional security theory. We build a moderated mediation model to examine the mediating effect of rejection sensitivity on the relationship between interparental conflict and adolescent IA and whether this indirect pathway is moderated by school connectedness. The model is shown in Figure 1.

## Materials and methods

### Participants

Participants were recruited from three junior middle schools (grades 7–9) in Guangdong Province, Southern China, through stratified and random cluster sampling in 2019. A total of 1,007 adolescents (51.84% females, *n* = 522) ranging in age from 12 to 15 (*M*_age_ = 13.16 years, *SD* = 0.67 years) participated in this study.

### Measures

#### Interparental conflict

Interparental conflict was measured the by Adolescents’ Perception of the Marital Conflict Scale (Chi and Xin, 2003). Participants were asked to answer seven items that investigate their perceptions of interparental conflict in families (e.g., When parents argue, they will lose their temper). All items were four-point scale. The average score was determined to reflect levels of interparental conflict, with higher scores indicating higher levels of interparental conflict. For the current measurement, the Cronbach’s alpha was 0.89.

#### School connectedness

School engagement was assessed by School Engagement Scale (Christenson et al., 2012). Adolescents were asked to report their engagement with other people and subjects (e.g., I feel safe and happy in school). The questionnaire was rated from 1 to 5, and the average score was calculated, with a higher score indicating high-level school engagement. For the current measurement, the Cronbach’s alpha was 0.68.

#### Rejection sensitivity

Rejection sensitivity was measured using the rejection sensitivity questionnaire (Li, 2007), adapted from the rejection sensitivity questionnaire by Downey and Feldman (1996). The questionnaire includes 18 items assessing interpersonal feelings (e.g., I am sensitive to others’ rejection), and six items are scored in reverse. Each item is rated on a five-point scale. The average score was calculated, with a higher score indicating high-level rejection sensitivity. For the current measurement, the Cronbach’s alpha was 0.86.

#### Internet addiction

Internet addiction was measured by a nine-item scale adapted from the Internet gaming disorder questionnaire (Pontes and Griffiths, 2015). Participants indicated how often they feel dependent on the Internet (e.g., “Do you systematically fail when trying to control or cease your internet use?”) on a three-point scale ranging from 1 (never) to 3 (often). Responses across the nine items were averaged, with higher scores reflecting a higher tendency to IA. In the current study, the measure demonstrated good reliability; the Cronbach’s alpha was 0.74.

### Statistical analyses

First, we used SPSS 26.0 to inspect descriptive statistics and correlations among variables. Next, we used PROCESS macro version 3.3 (Model 7) for SPSS (Hayes and Little, 2018) to test the mediation and moderation effects. These tests were conducted using pathway analysis with maximum likelihood estimation and bootstrapping with 5,000 iterations to estimate the 95% confidence intervals. To determine the presence of common method variance, Harman’s one-factor test was performed following the approach outlined by previous researchers (Lee et al., 2011). All participants’ self-report variables were entered into the exploratory factor analysis and the first factor accounted for 13.76% of the total variance, respectively, which was well below the threshold of 40%. Therefore, there was no common method bias.

## Results

### Preliminary analyses

Table 1 displays the means, standard deviations, and correlation coefficients for all variables. The results showed that interparental conflict was significantly, positively correlated with both rejection sensitivity. And both interparental conflict and rejection sensitivity positively relate to IA.

**Table 1:** Descriptive statistics and correlations for all variables.

Variables	1	2	3	4	5	6
1. Gender	−					
2. Age	0.06^*^	−				
3. IPC	−0.06^*^	−0.02	−			
4. RS	−0.20^***^	0.01	0.19^***^	−		
5. SC	0.06	0.04	−0.05	−0.03	−	
6. IA	0.07^*^	0.02	0.22^***^	0.29^***^	−0.01	−
*Mean*	0.48	13.17	1.72	3.04	3.70	1.18
*SD*	0.50	0.67	0.71	0.43	0.90	1.23

*N* = 1,007, gender was dummy coded such that 1 = male, 0 = female. IPC = interparental conflict, RS = rejection sensitivity, SC = school connectedness, IA = Internet addiction.

* *p* < 0.05;

*** *p* < 0.001.

### Testing for the moderated mediation model

**Figure 2:**
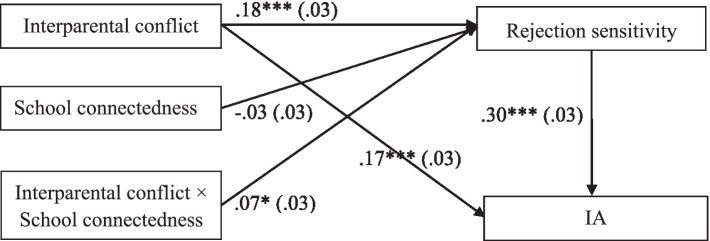
Model of the moderating role of school connectedness in the indirect relationship between interparental conflict and IA. Values are standardized coefficients. ^*^p < 0.05, and ^***^p < 0.001.

First, both of Model 1 (Rejection sensitivity as outcome variable, *R^2^* = 0.08, *F* = 17.12, *p* < 0.001) and Model 2 (IA as outcome variable, *R^2^* = 0.13, *F* = 38.10, *p* < 0.001) are significant. The results showed that interparental conflict positively relates to rejection sensitivity (*β* = 0.18, *t* = 5.55, *p* < 0.001), and rejection sensitivity positively relates to IA (*β* = 0.30, *t* = 9.28, *p* < 0.001). Moreover, the residual effect of interparental conflict on IA was significant (*β* = 0.17, *t* = 5.48, *p* < 0.001). Bootstrapping analyses indicated that rejection sensitivity significantly partially mediated the relation between interparental conflict and adolescent IA (indirect effect = 0.05, *SE* = 0.01, 95% CI [0.02, 0.08]), the mediating effect account for 22.73%. While controlling the effect of gender and age, results also display that girls will report more rejection sensitivity (0 = girls, 1 = boys, *β* = −0.38, *t* = −6.21, *p* < 0.001) and boys have higher level of IA (*β* = 0.28, *t* = 4.63, *p* < 0.001), age has no effect in each model (Figure 2).

**Figure 3:**
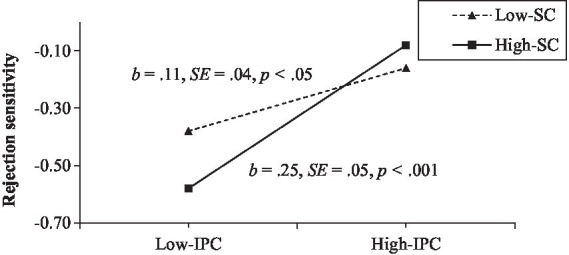
Rejection sensitivity among adolescents as a function of interparental conflict and school connectedness.

Moreover, interparental conflict and school connectedness have significant interactive effect on rejection sensitivity (*β* = 0.07, *t* = 2.22, *p* < 0.05). Given the interaction effect of interparental conflict and school connectedness on rejection, sensitivity was significant; we conducted a simple slopes test. As depicted in Figure 3, the positive association between interparental conflict and rejection sensitivity was significantly stronger among adolescents with higher school connectedness (1 *SD* above the mean: *β* = 0.25, *t* = 5.23, *p* < 0.001) than among adolescents with lower school connectedness (1 *SD* below the mean: *β* = 0.11, *t* = 2.58, *p* < 0.05).

Finally, results indicated that the indirect link between interparental conflict and IA *via* rejection sensitivity was stronger among adolescents with high school connectedness (indirect effect = 0.07, *SE* = 0.02, 95% CI [0.04, 0.11]) than among adolescents with low school connectedness (indirect effect = 0.03, *SE* = 0.01, 95% CI [0.01, 0.06]).

## Discussion

Our results indicate that interparental conflict is associated with adolescent IA and the results support hypothesis 1; however, we found counterintuitive results about the moderating effect of school connectedness. Besides, girls show higher rejection sensitivity (Koster et al., 2018) and boys show higher IA (Li et al., 2019) which consist with previous research; however, the effect of age is not significant that the *SD* of age is small so age may not impact adolescent rejection sensitivity and IA. These findings contribute to a deeper understanding of the development and influence factors of adolescent IA.

Consistent with other research that studies the deleterious influences of interparental conflict (Wu et al., 2016; Li et al., 2021); our research also found that interparental conflict was positively related to adolescents’ IA. The attachment theory and emotional security theory could explain our results (Bowlby, 1982; Davies and Cummings, 1994), interparental conflicts are stressful for adolescents and will harm their emotional and psychological security which let them to form an insecure attachment style, high interparental conflict adolescents may turn to the Internet for a sense of security (Wang L. X. et al., 2021). From the opposite perspective, positive family factors are important in understanding IA, parental monitoring, parental care, and communication are significantly negatively related to excessive Internet use (Faltynkova et al., 2020), which contrasts the harm of parental conflict from a positive perspective.

As we expected, rejection sensitivity significantly mediated the relationship between interparental conflict and adolescent IA. This mediation process is consistent with attachment theory in that a conflict environment may engender an insecure working model that they may always doubts and anxiety about whether others will accept and support them (i.e., rejection sensitivity); however, they will also spend a lot of internal resources to curb these anxieties (Lux and Walper, 2019). When adolescents spend too many internal resources on these meaningless anxieties, it is more difficult for them to control their maladaptive behavior (i.e., IA; Steinberg et al., 2006). Meanwhile, consistent with recent studies (Li et al., 2021), in a conflict family environment, parents may not compartmentalize their family roles as partners and co-parents, and their anger, stress, and rejection may spill over and affect their children (Demircioğlu and Köse, 2021), which made them more likely to rely on the Internet to escape from this stressful reality (Zhou et al., 2017). It is worth noting that rejection sensitivity just partly mediates the relationship between interparental conflict and IA. Thus, there might have another possible mechanism which worth investigating in future research. Our findings provide the first empirical study of rejection sensitivity as the vital mediating factor for the association between interparental conflict and adolescents’ IA.

Furthermore, each of the links in the mediation model is noteworthy. First, consistent with earlier research, our findings support that interparental conflict is direct positive with adolescent rejection sensitivity. Interparental conflict directly generates negative parenting (e.g., elevated rejection, coercion, and psychological control) and the parent’s negative emotions and behavior would spill over to the child (Krishnakumar and Buehler, 2000). And then, negative parenting leads to adolescents’ depressive symptoms and social anxiety symptoms, these are foundations of early adolescents’ rejection sensitivity (Rudolph and Zimmer-Gembeck, 2013). Second, our findings showed that adolescents’ rejection sensitivity positively relates to IA. Youth who are sensitive to rejection may not perceive others’ affection and may not feel a sense of belonging and security, and adolescents may turn to the Internet to compensate for their fundamental need for connection (Ryan et al., 2006). Internet contact may be a substitute for real-world relationships, and adolescents with high rejection sensitivity may use the Internet to escape from daily life to protect themselves from rejection. Research of Weinstein et al. (2016) support this assertion. The researchers found that online interaction is not only psychologically safer but also more convenient than real-life interaction. In summary, we indicate that rejection sensitivity can be a link between interparental conflicts and adolescent IA (Liu et al., 2020).

### Unexpected protective effect of school connection

To our surprise, our results did not conform to the protective-stabilizing model. The results indicated that adolescents with high school connectedness show a higher vulnerability to interparental conflict and report more rejection sensitivity in a high level of interparental conflict. Under the low parental conflict, the protective effect of school connection can be explained by Ecological Theory (Bronfenbrenner and Morris, 2006). We suggest that adolescents who build good connections with teachers and classmates in the school system will show higher self-esteem (Peng et al., 2019) and psychological security and report lower rejection sensitivity (Oldfield et al., 2018), individuals with high self-esteem and strong interpersonal security are not easy to distort their neutral signal into rejection (Jia et al., 2018). In contrast, school connectedness principally reflects the subjective relationship with peers and teachers; however, the influence of parents is profound and may override the effect of school connections. When the interparental conflict reaches a high level, the negative effect may not be ameliorated by a personal asset (Liu et al., 2016).

Therefore, we consider school connectedness as a double-edged sword for adolescents with high interparental conflict. When adolescents do not feel connected to their parents, they turn to other contexts to pursue other sources of bonding (Bretherton, 2005) which makes them pay more attention to the school environment. Although adolescents exposed to high interparental conflict may want to bond with others, they may have difficulties in constructing relationships because of their schema of anxiety and doubt, they are more likely to be rejected (Davies et al., 2018). Meanwhile, establishing multiple connections means more exposure to others’ neutral responses, but for sensitive adolescents with high interparental conflict, this may let them frequently and erroneously perceive rejection information that strengthen their rejection sensitivity (Li et al., 2021). Thus, school connectedness could show its positive edge for low interparental conflict individuals because they can truly get support from the school environment but show it is a negative edge for high interparental conflict adolescents because conflict family environment undermined their social skills but increased their social desire, the more school connectedness they wanted, the more rejection they felt.

We distinguished different patterns of the protective effect of school connectedness; the insights of these patterns help us understand the limitations and benefits of school connectedness while adolescents face family risky environments. Present results highlight the harmfulness of interparental conflict and should form targeted preventive interventions for adolescents that suffer from interparental conflict.

### Limitations and implications

Our research has several limitations. The cross-sectional research cannot reveal the causality or directionality of the associations we identified. Although we have focused on interparental conflict as the beginning of the risk process, adolescent IA may also result in interparental conflict (Ko et al., 2015). And interparental conflict consists of destructive and constructive conflict and it is important and interesting to further investigate the effect of constructive conflict on IA. A longitudinal design is a better way to test the directionality of these relationships. Secondly, we used adolescent self-reports to collect information, and although this method provides important information, multiple reporting sources can give us a more objective assessment. And the generalizability of the results needs to be tested. Further research is needed to determine if the findings apply in other countries or high risky samples. The last point is that in the study of the impact of family conflict on the development of adolescents, it may be more ecologically valid to consider multiple types of conflict and grasp the overall state of the family system (Cummings et al., 2015).

Despite these limitations, our study has some meaningful implications for high school students’ Internet addiction. First, our findings suggest that school connectedness still has protective effect while the low level of interparental conflict, therefore, educators should establish emotional connection with students and strive to build a harmonious class atmosphere to reduce students’ sensitivity to interpersonal insecure information. We also found mediation mechanisms between interparental conflict and early adolescent Internet addiction, which suggest that rejection sensitivity is an important internal mechanism, so cultivating students’ sense of secure emotion and guiding them to use adaptive coping strategies may be an effective way to prevent adolescents in high parental conflict environment from falling into Internet addiction. Lastly, since school connectedness has lost its protective role in high interparental conflict, we suggest that intervention can be directly applied to family parenting problems. Family-based cognitive-behavioral therapy has been proved that it can be used as an effective intervention to reduce the externalizing problems of adolescents (Sullivan et al., 2018), and this may be an effective way to reduce Internet addiction of adolescents in high interparental conflict environment.

### Conclusion

Our research is the first process-oriented study that proves rejection sensitivity as a mediator of the relationship between interparental conflict and IA. Furthermore, the mediating effect was moderated by adolescents’ school connectedness. Specifically, adolescents with high school connectedness showed higher vulnerability in the relationship between interparental conflict and rejection sensitivity, which give a valuable conclusion to reviewing the protective effect of school connectedness.

## Data availability statement

The raw data supporting the conclusions of this article will be made available by the authors, without undue reservation.

## Ethics statement

The study was conducted according to the guidelines of the Declaration of Helsinki, and approved by the Ethics in Human Research Committee of the Department of Psychology, Guangzhou University (protocol code: GZHU2019012, and date of approval: 2019/05/27).

## Author contributions

ZT, XZ, ZW, CY, and WZ contributed to the conception and design. CY and ZT performed material preparation, data collection, and analysis. ZT and XZ wrote the first draft of the manuscript, and all authors commented on previous versions of the manuscript. All authors contributed to the article and approved the submitted version.

## Funding

This work was supported by the “The role of executive function in sound interference reading: Evidence from eye movement studies” from the Humanities and Social Science Fund of Ministry of Education of China (20YJC190024).

## Conflict of interest

The authors declare that the research was conducted in the absence of any commercial or financial relationships that could be construed as a potential conflict of interest.

## Publisher’s note

All claims expressed in this article are solely those of the authors and do not necessarily represent those of their affiliated organizations, or those of the publisher, the editors and the reviewers. Any product that may be evaluated in this article, or claim that may be made by its manufacturer, is not guaranteed or endorsed by the publisher.
